# The Emergence of Virtual Tumor Boards in Neuro-Oncology: Opportunities and Challenges

**DOI:** 10.7759/cureus.25682

**Published:** 2022-06-06

**Authors:** Chukwuyem Ekhator, Santosh Kesari, Ramya Tadipatri, Ekokobe Fonkem, Jai Grewal

**Affiliations:** 1 Medicine, New York Institute of Technology, College of Osteopathic Medicine, Glen Cove, USA; 2 Translational Neurosciences, Pacific Neuroscience Institute, Santa Monica, USA; 3 Neurology, Barrow Neurological Institute, Phoenix, USA; 4 Neuro-Oncology, Baylor Scott & White Medical Center - Temple, Phoenix, USA; 5 Neuro-Oncology, Mount Sinai South Nassau Hospital, Oceanside, USA

**Keywords:** glioblastoma, neurology, neuro-oncology, cns tumor, virtual tumor board

## Abstract

Background

Virtual tumor board (VTB) platforms are an important aspect of cancer management. They enable easier access to a multidisciplinary team of experts. To deliver high-quality cancer care, it is necessary to coordinate numerous therapies and providers, share technical knowledge, and maintain open lines of communication among all professionals involved. The VTB is an essential tool in the diagnosis and treatment of brain cancer. For patients with glioma and brain metastases, multidisciplinary tumor board guidelines should guide diagnosis and therapy throughout the course of the illness. VTBs are an emerging resource across various cancer care networks in the United States.

Methodology

We performed a systematic search of all VTBs incorporating a platform designed for this specific role. We reviewed the records of the Genomet VTB, the Medical University of South Carolina (MUSC) VTB, and Xcures VTB. Summary data examined included the year of launch, demographics, characteristics of cases, average response time, advantages, and how they handle protected health information.

Results

Overall, 30% of VTBs examined were launched in 2017. All had a Health Insurance Portability and Accountability Act-compliant online environment. On a review of Xcures records, the median age of the female patients was 57 years and the median age of the male patients was 55 years. The data showed that 44% (4.4 out of every 10 patients) with a confirmed treatment chose the VTB integrated option. Overall, 76% of patients in the Xcures registry had primary central nervous system tumors, with at least 556 patients in the tumor registry which included 46% glioblastoma cases (96% primary, 4% secondary). In the MUSC VTB project, 112 thoracic tumor cases and nine neuro-oncology cases were reviewed. The tumor board met weekly, and the average response time was within 24 hours of case review and presentation. The Genomet VTB de-identifies all patient information; this is a virtual platform primarily focused on neuro-oncology cases. Cases involved a median of five specialists most commonly neuro-oncologists, neurosurgeons, radiation oncologists, molecular pathologists, and neuroradiologists. The case review revealed an age range of six months to 84 years (mean age = 44.5 years), with 69.6% males and 30.4% females, 43.5% glioblastomas, 8.7% adenocarcinomas, and 8.7% infratentorial tumors. The average response time observed in all cases was ≤24 hours.

Conclusions

VTBs allow for quicker expert analysis of cases. This has resulted in an accelerated number of cases reviewed with a shortened communication time. More studies are needed to gain additional insights into user engagement metrics.

## Introduction

Virtual tumor board (VTB) platforms are an important aspect of cancer management. They enable easier access to a multidisciplinary team of experts. To deliver high-quality cancer care, it is necessary to coordinate numerous therapies and providers, share technical knowledge, and maintain open lines of communication among all professionals involved. The VTB is an essential tool in the diagnosis and treatment of brain cancer. For patients with glioma and brain metastases, multidisciplinary tumor board guidelines should guide diagnosis and therapy throughout the illness course. VTBs are an emerging resource across various cancer care networks in the United States. Standard and effective healthcare delivery has become more important as the number and complexity of cancer cases have grown exponentially over the last several decades [1]. VTBs provide a platform for experts from various fields to get together and discuss how to best implement treatment protocols [2].

Coordinating numerous therapies and providers, sharing technical knowledge, and maintaining open lines of communication among all professionals involved are crucial for high-quality cancer care [3,4]. According to Gross’s definition of hospital tumor boards, which first appeared in 1987 [5], tumor boards are multidisciplinary teams of doctors who meet regularly to review cancer cases in order to improve the care of cancer patients in their community by exchanging information between participating physicians. The number of tumor boards has expanded tremendously since then, notably in the past 10 years, as their effectiveness in increasing diagnostic accuracy, adherence to clinical practice standards, and clinical outcomes has been proven.

In addition, VTB consultations enhance cancer patients’ access to the most recent cancer management plans and offer a tool for evaluating the quality of professional treatment provided by healthcare providers [6]. There is a wide range of professionals working together on a tumor board, ranging from thoracic surgeons to medical oncologists, radiologists, pulmonologists, pathologists, and molecular scientists. Nuclear medicine, nutrition, palliative and rehabilitation medicine, patient advocacy, research nursing, or other experts may be included in the expanded board [7]. In academic institutions, students and postdoctoral fellows may also be included [8].

The coronavirus disease 2019 (COVID-19) pandemic has changed the way patients are approached [9]. It is becoming more challenging to obtain a comprehensive health consultation. Therefore, the adoption of telemedicine such as VTBs may prevent any delay in the modification of care coordination in this moment of crisis [10]. Additionally, the notion of virtualization is being included in the design of cancer treatment because it may reduce geographic boundaries and simplify clinical communication and decision-making. However, videoconferencing technology, aided by the COVID-19 pandemic, has permitted the formation of VTBs in many cancer institutes and hospitals [11,12]. Meetings via the internet allow healthcare practitioners to work together more efficiently by reducing the time spent on travel as well as other associated costs [12,13].

A multidisciplinary team approach to diagnosis, therapy, and follow-up care in neuro­-oncology is critical due to the complexity of the disease. Oncological staging and treatment approaches are constantly evolving, and a multidisciplinary meeting is becoming more important [14]. As a quality checkpoint, the primary goal of these meetings is to guarantee that each case is thoroughly evaluated, regardless of whether it is pretreatment, during treatment, or posttreatment. Treatment planning, clinical trial enrolment, care coordination, management of treatment problems, evaluation of the disease response, recurrence monitoring, and survivorship outcomes are all part of this process [15].

Pretreatment assessment [16], accurate staging [17,18], and timely treatment [19-21] may be improved by the implementation of VTBs. Other factors may include disease-specific factors and overall survival [22-24]; however, there is some debate around this. Academic institutions are starting to analyze the quality metrics of these VTBs in relation to guideline adherence and clinical outcomes as the use of these meetings has become more common and this approach has become the standard of therapy [23].

VTBs have been implemented in various clinical settings for better evaluation of different cancers, including those of the lungs [13], liver [25], breast [11], gastrointestinal tract [11], and head and neck [26], in the last decades where the implementation of these board meetings resulted in enhanced referral coordination [27], shorter diagnostic and treatment latencies [13,25,27], increased prevalence of board evaluations [25], and decreased patient and clinician transport burden [25,27].

The VTB is an essential tool in the diagnosis and treatment of brain cancer. For patients with glioma and brain metastases, multidisciplinary tumor board guidelines should guide diagnosis and therapy throughout the illness course. For a tumor board in neuro­-oncology, there seems to be a paucity of information in the medical literature. This may be due in part to the specific training required for neuro-oncologists, neuroradiologists, and pathologists in this discipline [28-30].

To better serve patients with cancer, VTBs have developed over the years into more collaborative structures with teams that focus on rehabilitation, psychological well-being, and long-term care. Patients may also be present at these sessions, and it is important to have their input at each stage of the therapy. In addition, the members of the tumor boards share treatment choices and clinical responsibilities. Technology has made it simpler for VTB members to collaborate through a secured interface precluding the need for a face-to-face meeting [31]. The objective of this study is to provide a summary of a systematic review of VTBs and their implementation in neuro-oncology.

This article was previously presented as a meeting abstract at the 2022 American Academy of Neurology (AAN) Annual Scientific Meeting on April 5th, 2022.

## Materials and methods

We performed a systematic search of all VTBs incorporating a platform designed for this specific role. We reviewed the records of the Genomet VTB, the Medical University of South Carolina (MUSC) VTB, and Xcures VTB. Summary data examined include the year of launch, demographics, characteristics of cases, average response time, advantages, and how they handle protected health information.

## Results

On a review of Xcures records, the median age of female patients was 57 years and the median age of male patients was 55 years. The data showed that 44% (4.4 out of every 10 patients) with a confirmed treatment chose the VTB integrated option. Overall, 76% of patients in the Xcures registry had primary central nervous system (CNS) tumors. There were at least 556 patients in the tumor registry which included 46% glioblastoma cases (96% primary; 4% secondary), 13% diffuse midline glioma with *histone H3 lysine27-to-methionine* (*H3K27M*)-mutant cases, 5% diffuse intrinsic pontine glioma cases, 3% astrocytoma and diffuse astrocytoma cases, 3% anaplastic astrocytoma cases, 3% glioma and diffuse glioma cases, 1% anaplastic oligodendroglioma cases, and <1% gliosarcoma, oligodendroglioma, pilocytic astrocytoma, anaplastic pleomorphic xantoastrocytoma, fibrillary astrocytoma, oligoastocytoma, and pleomorphic xanthoastrocytoma cases. Tumor locations were distributed as 33% frontal, 19% parietal, 26% temporal, 4% occipital and 3% multifocal, 3% temporoparietal, 3% frontotemporal and thalamus, 2% frontoparietal, 1% parietal occipital, bifrontal, and corpus callosum, and <2% others.

In the MUSC VTB project, 112 thoracic tumor cases and nine neuro-oncology cases were reviewed. The tumor board met weekly, and the average response time was within 24 hours of case review and presentation. Patient information was uploaded through a secured patient portal access. Patient demographics were not recorded.

The Genomet VTB de-identifies all patient information; this is a virtual platform primarily focused on neuro-oncology cases. Cases involved a median of five specialists most commonly neuro-oncologists, neurosurgeons, radiation oncologists, molecular pathologists, and neuroradiologists. Participants had an average of ≥257 cases reviewed. Case review revealed an age range from six months to 84 years (mean age = 44.5 years), with 69.6% males and 30.4% females, 43.5% glioblastoma cases, 8.7% adenocarcinoma cases, 8.7% infratentorial tumor cases, and <5% each of pineoblastoma, melanoma, hemangioblastoma, and pilocytic astrocytoma cases. The average response time observed in all cases was ≤24 hours (Table 1).

**Table 1:** Summary of virtual tumor board characteristics.

Virtual tumor board	Xcures and cancer commons	Genomet	Medical University of South Carolina
Year of launch	2019	2017	2011
Approximate number of cases	≥556	257	112 thoracic and 9 neuro-oncology cases
Summary	The median age of female patients was 57 years and of male patients was 55 years. Overall, 2.44% of patients preferred the virtual tumor board integrated treatment option	Each case reviewed involved a median of five specialists most commonly neuro-oncologists, neurosurgeons, radiation oncologists, molecular pathologists, and neuroradiologists	Provides specialty consultation remotely for patients with brain tumors
Protected health information handling method	Health Insurance Portability and Accountability Act-compliant online environment		
Advantages	Reduced tumor board preparation time by more than 90%. Patients receive the benefit of multispecialty consultation. Integrated case-by-case expert analysis and shortened communication and response time. Improved productivity with data and documentation		

## Discussion

The use of tumor boards, as the benchmark in cancer care choice, has been extensively adopted. As handling cancer takes a lot of time and effort, and the prevalence of cancer is on the rise, this has put a lot of pressure on cancer treatment facilities, which are already stretched thin. As tumor care becomes more personalized, the formation of specialist tumor boards, such as neuro-oncological tumor boards, looks to be a worthy endeavor [32,33].

In neuro-oncology, VTBs have demonstrated advantages in both treatment and clinical outcomes. Other advantages include the opportunity for physicians to receive ongoing education and training, as well as the opportunity for them to reflect on their treatment strategies on a continuous basis. In addition, these VTBs have the potential to involve patients in clinical trials [29,30,34].

In this study including three VTBs, we found variability in the boards with respect to data regarding the demographics, characteristics of cases, average response time, and the way they handled protected information. Complex diagnostic information and treatment choices need technologies that assist organized codification, visualization, and interpretation of clinical data. Consequently, several academic fields, including psychology, improvement science, and organizational science, are producing more research on how to enhance tumor boards’ implementation. Even in more complicated instances, the advantages of VTBs become evident, leading to adjustments in treatment strategies and better results. Smart systems that can properly integrate, analyze, and comprehend clinical data are becoming more important as the amount and complexity of available data keep growing [35].

Henderson et al. [36] reported a case of a five-year-old boy with a history of vomiting for two months which was followed by headaches that worsened over time. There were no neurological deficits on examination, but the magnetic resonance imaging (MRI) showed a cystic lesion with obstructive hydrocephalus. Subject to the location of the tumor and associated obstructive hydrocephalus, the attending surgeon desired input from the tumor board. Even though the condition of hydrocephalus did not require assistance, the treatment approach (ventriculoperitoneal shunting versus endoscopic third ventriculostomy), the timing of treatment in anticipation of possible craniotomy, and the approach adopted in the event of surgical treatment all had risk and benefit considerations. With the help of a VTB (Genomet, New York), the de-identified case profile was developed online and communicated with a voluntary network of neurosurgeons located in the United States through an online email connection. Within 12 hours, three surgeons replied with clinical feedback on the case. In accordance with their suggestions, the patient underwent an endoscopic third ventriculostomy and was released home the next day without experiencing any headaches. After a few months, the patient intended to return for tumor excision. The study presents some cases from a neuro-oncological viewpoint where the implementation of VTBs was mandatory to personalize treatment by involving experts from different fields in the VTB.

Case studies discussed in VTBs

Case 1

**Figure 1:**
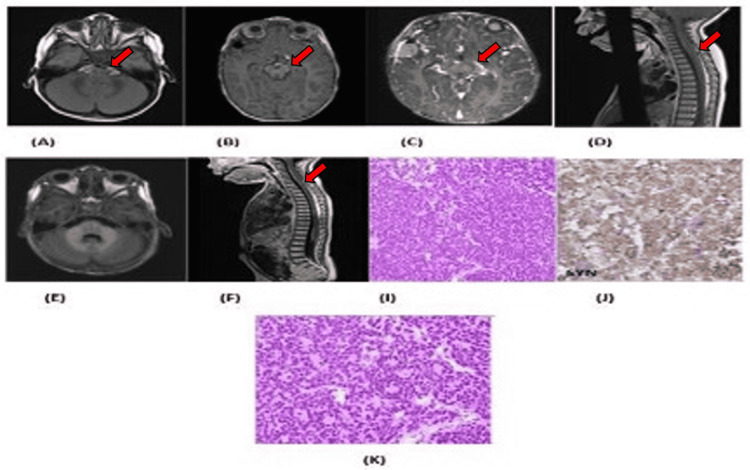
(A) Brain MRI at diagnosis showing a suprasellar mass (red arrow). (B) Best response to first-line therapy: brain MRI (red arrow) showing suprasellar mass regression. (C) First recurrence brain MRI (red arrow). (D) First recurrence spine MRI (red arrow). (E) Best response to second-line therapy: brain MRI. (F) Second recurrence spine MRI. (I) H&E stain showing WHO grade 4 CNS embryonal tumor. (J) IHC showing strong synaptophysin expression. (K) H&E showing scattered rosettes. MRI: magnetic resonance imaging; H&E: hematoxylin and eosin; WHO: World Health Organization; CNS: central nervous system; IHC: immunohistochemical

A six-month-old, full-term male presented with three weeks of abnormal eye movements and one day of left periorbital swelling and clear ocular discharge. An examination demonstrated left cranial nerve (CN) III and VI palsies and left lateral eyelid swelling and ptosis. Imaging revealed a diffusion-restricted suprasellar mass without metastatic disease. A biopsy was undertaken (resection was deemed high risk) and demonstrated a CNS embryonal tumor (WHO grade 4) with somatic mutations in *retinoblastoma 1* (*RB1*) and *mutY DNA glycosylase* (*MUTYH*). Methylation analysis identified the tumor as a pineoblastoma group A/PB-RB1 subgroup (even though the tumor was not located in the pineal gland) (Figure 1). Initial treatment consisted of (1) one cycle per HEADSTART IV (cisplatin, cyclophosphamide, etoposide, IV methotrexate, and vincristine): complicated by bronchospasm (secondary to etoposide), pericardial effusion leading to tamponade physiology (led to heart failure and pulseless electrical activity (PEA) cardiac arrest requiring venoarterial extracorporeal membrane oxygenation (VA-ECMO)); imaging demonstrated a slight decrease in tumor size. (2) One cycle per ACNS0334 A (cisplatin, 50% dose-reduced cyclophamide, etoposide, and vincristine): complicated by thrombotic microangiopathy (secondary to cisplatin) leading to renal insufficiency, hypertensive urgency, severe hemolytic anemia and thrombocytopenia, and pulmonary edema requiring bilevel positive airway pressure (BiPAP); imaging demonstrated a near-complete tumor response. (3) Nine cycles of metronomic therapy (PO cyclophosphamide, etoposide, and temozolomide alternating with PO celecoxib and isotretinoin; q4 week IT topotecan).

The first recurrence was observed after nine cycles (six months) of metronomic therapy, with imaging demonstrating local and distant recurrence. Salvage treatment was initiated with carboplatin, etoposide, and ifosfamide and was complicated by seizures (likely secondary to the metastatic tumor but potentially triggered by ifosfamide neurotoxicity); hence, ifosfamide was replaced with cyclophosphamide for the following two cycles. Imaging after cycle two demonstrated a near-complete tumor response. Even though the plan was to collect autologous stem cells, cells could not be mobilized. The second recurrence was observed after three cycles of salvage therapy, with imaging again demonstrating new diffuse leptomeningeal disease. Palliative treatment with PO etoposide was initiated and then transitioned to gefitinib.

Several complications were associated with the case and the resection was deemed to be high risk because of complications. Second, the adverse outcome of treatments necessitated input from different experts, including neuro-oncologists, neurosurgeons, radiation oncologists, molecular pathologists, and neuroradiologists.

Case 2

There was another case of an 18-year-old male who developed progressive back pain and paraesthesia starting in April 2017. As this worsened, he underwent imaging in October 2017 and was found to have a thoracic spinal mass. At the time, he was ambulatory with mild foot weakness only. He underwent a surgical resection on October 5, 2017, and was diagnosed with a WHO grade 1 ganglioglioma. He subsequently developed syringomyelia, lost leg strength and his ability to walk, and developed bowel and bladder incontinence. He then underwent a second surgical operation on June 11, 2018. The diagnosis was updated to a high-grade WHO grade 4 glioma with the presence of the *H3K27M* point mutation. The neuronal elements previously described were no longer evident. He underwent radiation to the thoracic spine tumor from July 2, 2018, to August 5, 2018, receiving 1.8 Gy in 28 fractions. A molecular profile was ordered from the second surgical specimen.

He received one cycle of the combination of temozolomide and CCNU as initial therapy in August 2018. The molecular report became available on September 30, 2019. Due to the presence of *fibroblast growth factor receptor 1* (*FGFR1*) and *neurofibromatosis type 1* (*NF1*) mutations, temozolomide + CCNU was discontinued (without evidence of progression) and sorafenib monotherapy was initiated at 400 mg PO twice daily. The patient experienced clinical and radiographic benefits for approximately six months. After this time (March 11, 2019), there appeared to be imaging progression, and FOLFIRI (5-fluorouracil, irinotecan, folate) was added to sorafenib. He took FOLFIRI + sorafenib from July 10, 2019, to September 1, 2019. He presented for treatment recommendations in the setting of his molecular findings. He did not wish to consider surgical options. Neurologically, he had no leg strength (0/5) and impaired bowel and bladder function but progressively improved and was fully able to use his arms to transfer and perform self-catheterization. The patient underwent his first surgery of spinal cord tumor resection with extensive instrumentation in Norway. The patient was neurologically stable immediately postoperatively but later developed syringomyelia. The patient then underwent re-resection due to tumor progression. Pathological diagnosis was updated to high-grade glioma, *H3K27M*. Subsequently, radiation therapy was initiated with an external beam and was limited to the cervical and thoracic spine, with 50.4 Gy in 28 fractions, which resulted in stable disease followed by progression. After that, systemic therapy was started with temozolomide 200 mg/m^2^ for five days + CCNU 110 mg/m^2^ for one day, which led to stable disease, followed by a second systemic therapy course with sorafenib 400 mg BID, chosen due to the presence of *FGFR1* and *NF1* mutations, which resulted in partial response followed by progression. A third systemic therapy was started with sorafenib 400 mg BID + FOLFIRI, which resulted in stable disease followed by progression. The patient was resistant to topoisomerase inhibitors, sorafenib, and FOLFIRI, and he was not a candidate for surgical options. The patient’s tumor mutational burden score indicated eight mutations, and his Karnofsky Performance Status (KPS) score was 40. Molecular findings are summarized in Table 2.

**Table 2:** Summary of molecular findings identified in virtual tumor board cases. IDH: isocitrate dehydrogenase; MGMT: methylguanine-DNA methyltransferase; MSI-H: microsatellite instability-high; NF-1: neurofibromin-1

Molecular finding	Variant interpretation	Tissue tested
*H3K27M* mutation	Mutations in *H3F3A*, which encodes histone H3.3, commonly occur in pediatric glioblastoma. Additionally, *H3F3A K27M* substitutions occur in gliomas that arise at midline locations (e.g., pons, thalamus, spine); moreover, this substitution occurs mainly in tumors among children and adolescents	Spinal cord
NF1 deletion	Neurofibromin, the protein product of NF1, regulates the inactivation of the Ras pathway and acts as a tumor suppressor. Recent studies have identified somatic alterations in the gene encoding for neurofibromin (*NF1*) in a subset of glioblastoma (GBM), usually associated with the mesenchymal molecular subtype. NF1 loss was associated with worse overall and disease-specific survival in the lower-grade glioma, but not the GBM group in the Cancer Genome Atlas cohort. IDH1 or 2 mutations co-existed in lower-grade gliomas with NF1 loss (36%) but not in GBM	Spinal cord
MS1-H	No identifiable defects of mismatch repair. Microsatellite instability (MSI) is a pattern of hypermutation that occurs at genomic microsatellites and is caused by defects in the mismatch repair system. Mismatch repair deficiency that leads to MSI has been well described in several types of human cancer, most frequently in colorectal, endometrial, and gastric adenocarcinomas	
Un- methylated MGMT	MGMT (0-6 methylguanine DNA methyltransferase) is a DNA-repair enzyme which repairs the naturally occurring or therapeutically induced mutagenic DNA lesion 06-methylguanine back to guanine and prevents mismatch and errors during DNA replication and transcription. Tumor MGMT methylation is predictive of increased benefit from DNA damaging therapies, such as radiation therapy and temozolomide, as well as generally prognostic for survival. In large, randomized trials, GBM patients with MGMT-methylated tumors had improved prognosis (longer survival) than patients who are unmethylated	Spinal cord
IDH wildtype	*IDH1 R132H *point mutations are generally associated with secondary GBM and more indolent tumor behavior than tumors that are IDH1 wildtype. IDH1 status is a component of the WHO 2016 criteria for certain astrocytomas including GBM. IDH is an enzyme that normally converts isocitrate to alpha-ketoglutarate as part of the TCA cycle	

The repetitive progression of the disease after every treatment approach and the resistance to drugs required input from several experts on a VTB platform to personalize treatment according to the molecular findings.

Case 3

**Figure 2:**
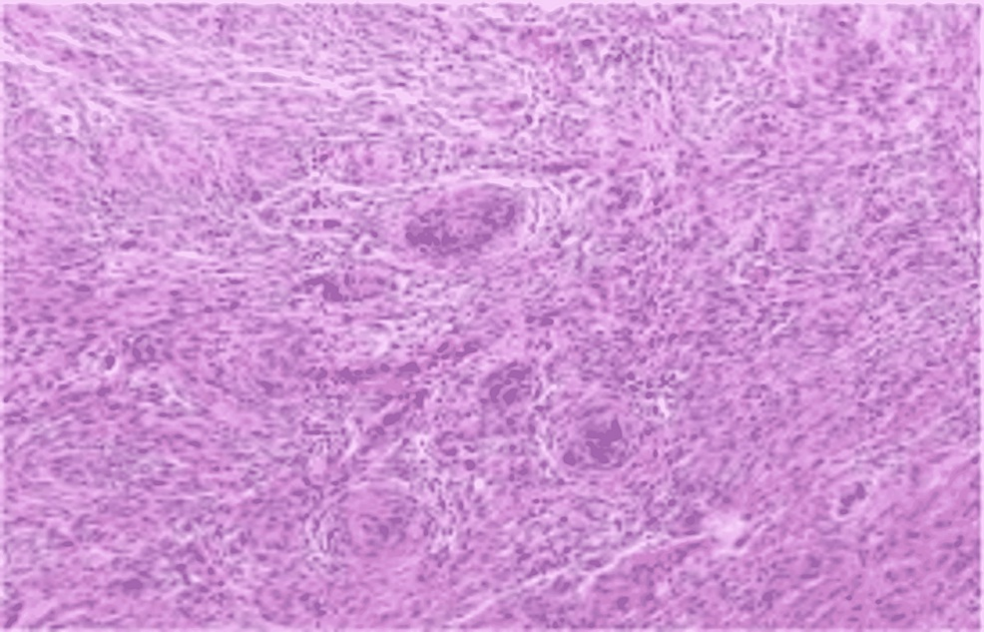
H&E stain showing WHO grade 1 meningothelial meningioma. H&E: hematoxylin and eosin; WHO: World Health Organization

Another case discussed by the online VTB Genomet was the case of a 56-year-old male with progressive, octreotide-positive meningioma who presented for medical salvage treatment options. He underwent two resections, intensity-modulated radiation therapy (IMRT), and two courses of stereotactic surgery (SRS). Chemotherapy with sunitinib was switched to bevacizumab due to intractable hand and foot syndrome. He had mild right leg weakness with a KPS score of 70. The patient underwent craniotomy and resection of left interhemispheric meningioma with no postoperative complications, followed by another surgery after four years for seizure and intractable cerebral edema and right leg weakness. Small wound dehiscence resolved by four weeks postoperatively. Pathology showed WHO grade 1 with low mitotic activity (<10%) (Figure 2). He was started on radiation therapy after three months with IMRT 5,600 cGy in 28 fractions, which was tolerated well and showed a partial response followed by progression. A second radiation therapy course was started with GammaKnife SRS receiving 16 Gy single fraction, which also resulted in partial response followed by progression. A third radiation therapy course was started with Novalis TX SRS receiving 22.5 Gy in five fractions, which resulted in stable disease followed by progression. He developed grade 4 hand and foot syndrome requiring discontinuation after treatment break, dose reduction, and unsuccessful re-challenge. There was a partial response followed by progression. A second systemic therapy course was started with bevacizumab 10 mg/kg every two weeks; eight cycles (14 days) were completed. He had stable disease after four cycles, but progression was observed. PEGylated interferon-alpha was not administered due to toxicity/adverse events and risk of injury.

Case 4

**Figure 3:**
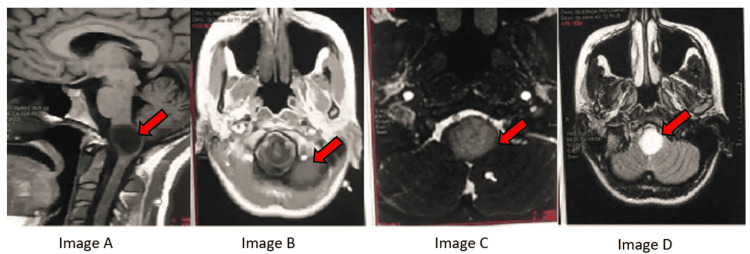
(A) Sagittal T1: hypointense cystic brain stem mass (red arrow). (B) Axial T1+C: nodular enhancement (red arrow). (C) Axial T2 FLAIR: iso/hyperintense (red arrow). (D) Axial T2: hyperintense (red arrow). FLAIR: fluid-attenuated inversion recovery

Another case presented was of a 28-year-old female who presented with headaches, dizziness, and visual impairment, followed by gait ataxia. MRI revealed an oval expansive formation in the brainstem, a hypointense signal in T1, isointense on fluid-attenuated inversion recovery (FLAIR), and hyperintense on other sequences with a nodular focus of contrast enhancement measuring 3.2 × 2.3 × 2.3 cm located in the brainstem at the level of the bulb medullary transition, compressing the cerebrospinal column at the level of the foramen magnum (Figure 3). There was no evidence of hyperperfusion. Biopsy showed pilocytic astrocytoma, WHO grade I, glial fibrillary acidic protein (GFAP)-positive; inconclusive synaptophysin; negative for neurofilament, isocitrate dehydrogenase (IDH1), and p53 mutation. Ki67 was 5%.

Subject to the findings and examination, only a biopsy was possible in this case and radiotherapy was contraindicated due to risk. Guidance from tumor board experts was needed with respect to the systemic therapy in this case.

Case 5

**Figure 4:**
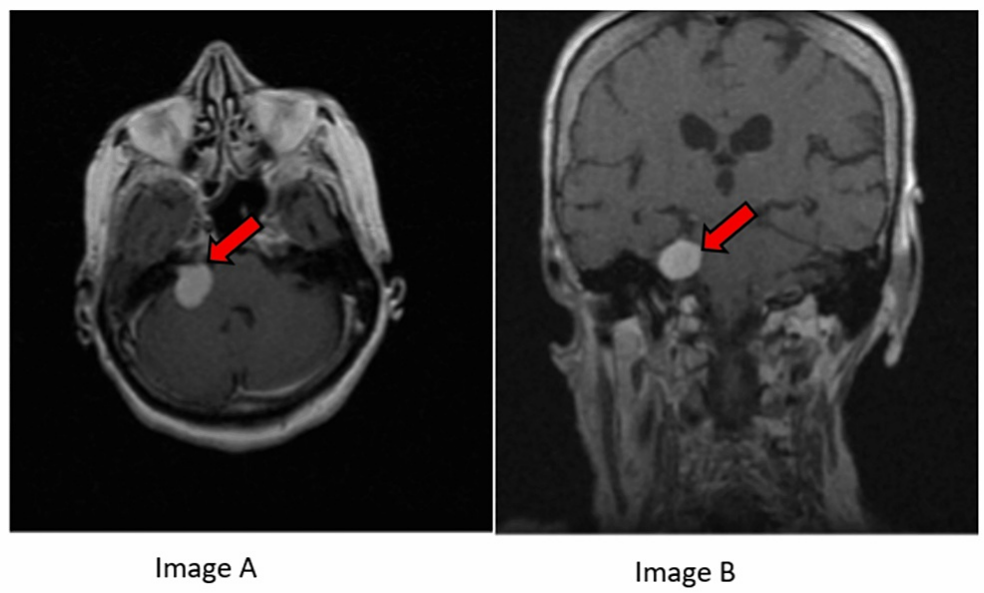
Axial radiological scan (A) (acoustic schwannoma at the cerebellopontine angle) and coronal radiological scan (B) (acoustic schwannoma at the cerebellopontine angle).

Another case was of an 84-year-old female who presented with subacute hearing loss, confirmed on audiometry. Brain imaging revealed a right cerebellopontine angle (CPA)-enhancing mass. The diagnosis was of an acoustic schwannoma of the brain, infratentorial, WHO grade 1. The patient underwent surgical resection of the right CPA mass, presumably acoustic schwannoma. Other therapies were rejected due to the risk of toxicity/adverse events and risk of injury. Comorbidities included kidney disease, hypertension, and diabetes, and the patient’s Eastern Cooperative Oncology Group (ECOG) Performance Score was 1. The patient’s axial and coronal scans are shown in Figure 4.

Case 6

**Figure 5:**
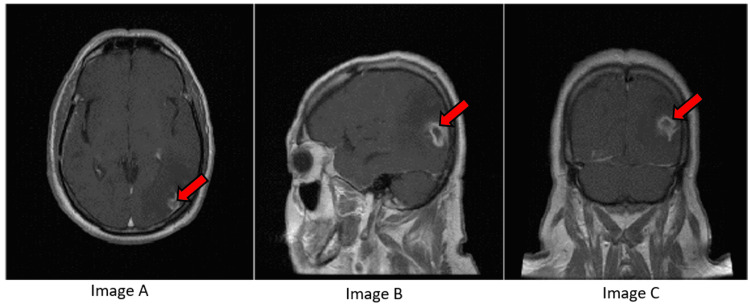
Radiological image showing a left occipital well-circumscribed mass (red arrow) in different sections outlined in A, B, and C (glioblastoma and IDH-wildtype). IDH: isocitrate dehydrogenase

Another case discussed was that of a 73-year-old female as a newly diagnosed patient with subtotal resected left occipital glioblastoma who presented for additional treatment recommendations. She was diagnosed with glioblastoma, IDH-wildtype, WHO grade 4, in the brain. She underwent subtotal resection of a left occipital glioblastoma, and there were no other complications. Comorbidities included kidney disease and a history of a liver transplant. The patient was resistant to alkylating agents and had a KPS score of 50. Her molecular findings are shown in Figure 5.

Opportunities and challenges in the implementation of VTBs

Implementation of VTBs in neuro-oncology has demonstrated effectiveness in unraveling certain treatment crossroads. All the cases presented above required input and consultation with a multidisciplinary tumor board. The complexities in the cases necessitated guidance with respect to each approach to be adopted, including surgery, radiation therapy, and systemic therapy. VTBs have been utilized effectively in various countries and specialties, including gynecologic oncology, since at least 2004, despite being novel in the context of neuro-oncology [37]. A study on the automation of VTBs reported a twofold increase in the number of cases evaluated each month. In addition, VTBs provided more possibilities than a lab report alone. In 503 of the 642 patients, VTBs allowed for genomic-based treatment choices to be made available to 78.6% of patients. In total, 229 and 80 patients received on-label and off-label therapy recommendations, respectively, based on proteomic data. Importantly, VTBs skipped therapy alternatives in 64% of individuals who had established resistance to earlier treatments.

Implementing a VTB program has been fraught with difficulties, including credible technical setup [13,27], expanded duration of virtual case presentations [1], scarcity of community-based caseloads [11], delays in obtaining supportive evidence [11], and the high cost of virtual information technology facilities [11,38]. Despite these obstacles, it seems that VTB participants in general either support it or find it equivalent to regular in-person meetings concerning effectiveness and efficiency [1,11,26]. The degree to which VTBs influence guideline compliance and clinical outcomes in contrast to a standard tumor board is yet to be determined by researchers. It will be possible to discern these links when more data on the development of VTB quality become available [12].

One downside of using a VTB is the absence of physical connection while discussing patient care issues with other members of the VTB. There is a consequent decline in the sense of brotherhood among physicians across specializations. Additionally, while utilizing videoconferencing software, it might be difficult to have engaged discussions with numerous speakers at once. Software issues, such as screen sharing and audio problems, were the most challenging to overcome, particularly during the initial VTB sessions held online. However, in future sessions, this was made more acceptable. Finally, some participants reported that it was more difficult to keep track of the list of patients mentioned during the VTB session than others [12].

The fundamental drawback of this study, which is also true of all surveys, is the inability to generalize the findings based on the sample of VTBs enrolled in the study. Another limitation of VTBs is obtaining metrics from individual VTBs as most of their metrics are unpublished. For a VTB to be successful and sustainable, it must go through a process in which various parts of the application are regularly examined, allowing for improvements before it can be widely used. Additional considerations for adoption include privileging, credentialing, and financial considerations, among other things.

## Conclusions

VTBs have allowed for quicker expert analysis of cases. This has resulted in an accelerated number of cases reviewed with a shortened communication time. Its implementation in neuro-oncology and other specialized fields will result in personalized healthcare provision in collaboration with experts from different disciplines. More studies are needed to obtain additional insights into user engagement metrics.
